# Investigating Multi-Level Semantic Extraction with Squash Capsules for Short Text Classification

**DOI:** 10.3390/e24050590

**Published:** 2022-04-23

**Authors:** Jing Li, Dezheng Zhang, Aziguli Wulamu

**Affiliations:** 1 School of Computer and Communication Engineering, University of Science and Technology Beijing, Beijing 100083, China; b20200337@xs.ustb.edu.cn (J.L.); zdzchina@ustb.edu.cn (D.Z.); 2 Beijing Key Laboratory of Knowledge Engineering for Materials Science, University of Science and Technology Beijing, Beijing 100083, China

**Keywords:** multi-level semantic extraction, capsule network, short text classification, deep learning

## Abstract

At present, short text classification is a hot topic in the area of natural language processing. Due to the sparseness and irregularity of short text, the task of short text classification still faces great challenges. In this paper, we propose a new classification model from the aspects of short text representation, global feature extraction and local feature extraction. We use convolutional networks to extract shallow features from short text vectorization, and introduce a multi-level semantic extraction framework. It uses BiLSTM as the encoding layer while the attention mechanism and normalization are used as the interaction layer. Finally, we concatenate the convolution feature vector and semantic results of the semantic framework. After several rounds of feature integration, the framework improves the quality of the feature representation. Combined with the capsule network, we obtain high-level local information by dynamic routing and then squash them. In addition, we explore the optimal depth of semantic feature extraction for short text based on a multi-level semantic framework. We utilized four benchmark datasets to demonstrate that our model provides comparable results. The experimental results show that the accuracy of SUBJ, TREC, MR and ProcCons are 93.8%, 91.94%, 82.81% and 98.43%, respectively, which verifies that our model has greatly improves classification accuracy and model robustness.

## 1. Introduction

Short text usually exists in different styles, such as micro-blog, chat messages, news topics, opinion comments and mobile phone text messages. Short text has strong sparsity and usually only contains a few to dozens of meaningful wrds. Therefore, it is difficult to extract effective feature words. In addition, short text exists in a large nuber of people’s lives and its update speed is fast. The Internet has accumulated an enormous amount of short text data because of the timely update and rapid spread of them [1], which requires a high speed in the processing and calculation of short text. A consideration of the above characteristics results in short text classification facing the following problems: the features of short text are limited, and the traditional vector space model based on entries leads to sparse vector space [2]. In addition, word frequency, word co-occurrence and other information cannot be fully utilized, which loses the potential semantic correlation between words. The irregularity of the short text makes irregular feature words and unknown words appear in the text, which cannot be realized by the segmentation dictionary. It leads to the inaccuracy of the traditional text preprocessing and text representation methods.

The capsule network is regarded as a new kind of neural network that can replace traditional neural networks in the future [3]. In other words, the capsule network can be defined as replacing neurons with capsules. The core concept of a capsule network is inverse rendering (IR). In comparison to rendering, IR deduces information about objects, including spatial geometric information based on images. The mission of the capsule network is to learn how to reverse rendering, which predicts the instance parameters of the image by observing the image. The initial application of the capsule network is mainly in the field of images, but its application in the text direction is relatively lacking. However, there are studies that still prove the effectiveness of the capsule network in text classification [4]. More importantly, the capsule network also showed a significant improvement in converting single-label text categorization to multi-label text categorization, compared to the strong baseline model.

The main contributions of this paper are listed as follows:

1. It proposes a new classification model from the aspect of short text representation, global feature extraction and local feature extraction. It uses convolutional networks to extract shallow features, and introducing a multi-level semantic extraction framework that includes the encoding layer, interaction layer and concatenation layer. It is combined with the capsule network to obtain high-level local information. It extracts short text semantics to maximize the possibilities within the limited text and improve the quality of the feature representation.
2. It explores the optimal depth of semantic feature extraction for short text classification based on a multi-level semantic framework.
3. Experiments were carried out on four public datasets and quantitative comparative experiments were carried out, and considerable results were obtained.

The paper is organized as follows. Section 2 illustrates the related work on short text classification and capsule-based text classification. Our proposed novel approach is presented in Section 3. In Section 4, we discuss the experiment result and the discussion. Finally, the conclusion and the future roadmap are presented in Section 5.

## 2. Related Work

**Figure 1:**
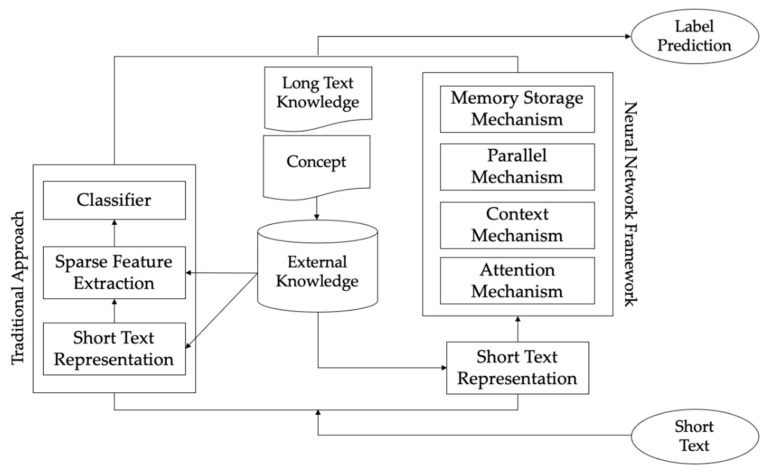
The pipeline of the traditional approach and neural network framework for short text classification.

Short text classification can be regarded as a task that selects the affiliation of short text categories according to specific categories [5,6]. Most short text classification methods are combined with a topic word or keyword extraction to find the core elements of short texts to perform the short text classification task. Figure 1 depicts a pipeline of short text classification and shows the relationship between the traditional approach and neural network framework for short text classification.

### 2.1. Short Text Classification based on Deep Learning

The deep learning method is popular because of its strong data processing and feature extraction abilities [7]. As we know, CNNs are often used to process computer virtual problems. For the text problem, it firstly produces text vector embedding, converts the text vector into a convolution layer, and the max-pooling layer then performs its role with the SoftMax output layer for the classifications. Hu [8] proposed the novel short text classification technique based on Twitter (social media), which mainly uses convolutional neural networks to perform feature engineering, and uses a support vector machine for classification. ABRNN [9] uses attention for short text classification with a recurrent neural network. To automatically filter the specific area tweets, it allows the network to separately weigh words in each tweet based on their varying importance. Then, the weights placed on each word are shown by using a heatmap. DE-CNN [5] greatly differs from a traditional CNN, which is the addition of context-relevant concepts. The specific embedding of every word is attained by BiGRU. With the help of Probase [10], the concept and word sets will be constructed, since a single word belongs to different concepts in a diverse text context.

The preponderance of the phenomenon of less processed data and more unprocessed data has been fully utilized in a proposed framework by storing text features and transferring information in the form of graphs [11]. This innovation mainly focuses on integrating all of the short text information, even though the additional text is for strengthening the text representation. Chen [12] also solved the problem of less annotated data in this classification task. A conditional independent model was designed to automatically produce the label, which was used to solve the problem of imbalanced data. Liu [13] proposed a multi-level attention combination network with the use of an external knowledge base that can effectively extract the context features. Therefore, this approach enriches the representation of short text, which resolves the text sparsity and ambiguity problem. Škrlj [14] constructed a new angle for feature construction, which is word taxonomies. The feature effectively improved the performance of the classifier, especially when the data was scarce. Feng [15] applied two layers of attention to parse the short text, and the proposed framework can be transferred to multi-label text classification tasks. 

### 2.2. The Capsule Network for Text Classification

**Figure 2:**
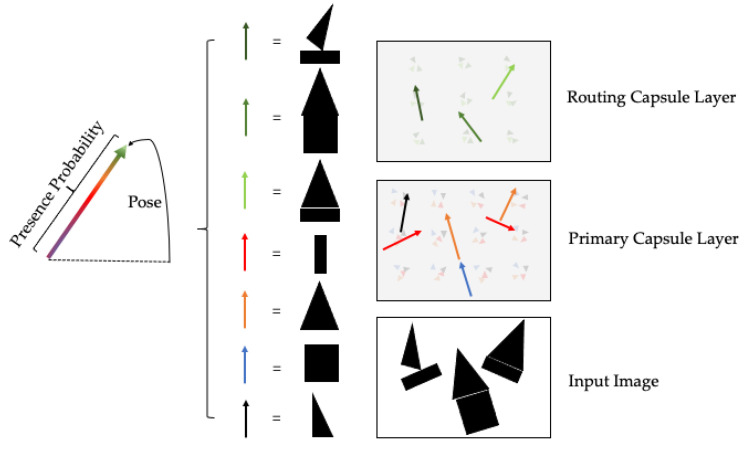
The application of the capsule network in the image area.

Deep learning algorithms with strong transferability factors, such as CNN, can be well applied to NLP tasks, but they cannot take into account the hierarchical relationship between local features. Capsule networks can not only train the model with fewer data, but can also process the ambiguity of the picture, since it is the first rise in the field of the image. Figure 2 shows the main construction of the capsules in the image task. In this kind of task, the capsule is different from the attention mechanisms, such as mutual attention learning [16] or adaptive attention learning [17], and it can be divided into basic and higher capsules, which are also called routing capsules. By testing the pose and presence probability of the specific part, the small and larger objects can be detected from these two kinds of capsules, respectively. In the text area, Sabour et al. [18] proposed the capsule network to improve the limitations of CNN feature extraction. Additionally, they updated the dynamic routing mechanism between the master and digital capsules to obtain a high-level representation. Zhao et al. [19] expressed the effectiveness of the capsule network in text classification by improving the stability of the DR update mechanism. The model uses the statistic feature for the first layer, then connects the capsules. It proved that the capsules can obtain better results when an interim exists in the text dichotomy and multi-classification. Jia [20] explored the capsule network model based on attention enhancement by combining syntactic analysis and sequence structure. Gangwar [21] also explored the model that uses GloVe to train the embedding of the text. The BiGRU acts as an interaction layer that connects with a primary capsule. It achieves better accuracy by using the text semantic features extracted by BiGRU without the import of extra data. Du [22] proposed utilizing capsules to build semantic information and clustered them by EM routing. Additionally, an attention mechanism was applied in the capsule selecting an algorithm to process the feature connection between the short terms and context. Zheng [23] designed a capsule-based model named HAC. First, a hierarchical structure features are formed by adopting the interactive information of a meticulously designed deep, extended CNN. Chen et al. [24] proposed a structure to link the long-level text information to short-level text sentiment analysis, which is named the Transfer Capsule Network. They made short-level text and long-level text data of sentence-level feature representation encapsulated into feature capsules. They performed experiments on the SemEval dataset, which showed the effectiveness of TransCap. Du et al. [25] used capsules on a short text sentiment analysis task. They proposed a hybrid network that can attain the text feature information, which is difficult to extract effectively. The interdependent features with long distances were attained by a bi-directional gated recurrent unit. Zhang et al. [26] resolved the problem of sentiment classification with capsule networks in the area of the domain scenario, with consideration of the language semantic laws. They also proposed a law network to integrate the language laws to enhance comprehensive sentence representation. A capsule-based pipeline network that aims to utilize the attention method for information extraction from high-level data was proposed [27]. It provides a system for short sentences to pledge strengthened supervisory control and information quality. Kim [28] conducted in-depth research on the application of capsule networks in text classification, and proposed a clear routing selection algorithm that effectively decreased the computer calculation of dynamic routing and the validity of the network was verified on several datasets.

## 3. Methodology

### 3.1. The Convolution Semantic Matrix Module (CSMM)

**Figure 3:**
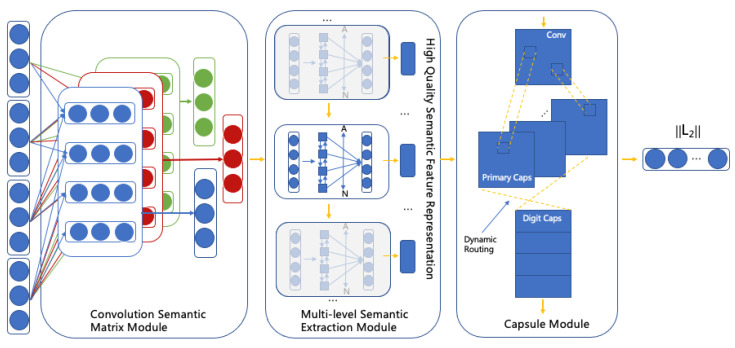
The proposed framework.

The convolutional neural network [29] has been widely applied in the image area because its excellent performance in this field lies in its feature extraction ability. It can gradually extract from low-level features, such as original image pixels to edges, corners and contours. Additionally, this particular hierarchical representation phenomenon exists not only in image data, but also in text, from word to word, phrase, sentence and paragraph. This process also reflects the phenomenon of feature hierarchy, so CNN is applied to the shallow semantic feature representation module of a short text in this framework. The application of CNN in our model is shown in Figure 3.

In the process of using a convolutional neural network, each row vector in the sentence vector is the representation of words, and the column vector is the splicing of sentence words [30]. The framework proposed in this paper applies a new multi-channel convolution structure to characterize the text. Firstly, the multi-channel input method is adopted to simultaneously calculate the respective features, and the feature map is generated through the respective continuous convolution, which constitutes the final feature vector.

Define the short text representation as $ST_{n\times d}$ = $s_{1}⊗s_{2}⊗\ldots ⊗\;s_{i}⊗\ldots ⊗\;s_{n};\;ST_{n\times d}$ is the matrix representation; n means the total length of the short text; d is the word embedding size; $s_{i}$ is the *i*th word in short text; and ⊗ represents the immediate semantic concatenation of the word. After feeding $ST_{n\times d}$ into the multi-channel convolution, the convolution layer generally chooses convolution kernels of various sizes to perform the convolution operation for the input matrices, so as to obtain more semantic unit information. The feature operation formula of this convolution layer is as follows:

(1) $$\sum_{1:n-h+1}x_{i}=\left(w\cdot ST_{i:i+h-1}\right),$$

In the formula above, w is the convolution kernel matrix, $ST_{i:i+h-1}$ represents the lines *i* through *i* + *h* − 1 of the text matrix, and the output is the feature matrix x((n−h+1)×k),

(2) X=f((n−h+1)×k+b),

f is the activation function, a linear function of the unilateral inhibition ReLU, and b is offset item.

### 3.2. The Multi-Level Semantic Extraction Module (MlSEM)

To solve the problem that semantic features of short texts are difficult to be extracted, we design a novel network MlSEM. In the present study, the feature vector obtained in the previous step is deeply mined and represented, and the best depth of MlSEM is explored according to the accuracy of the classification task. It consists of six similar semantic extraction modules and every module includes three parts, which are the encoding layer, interaction layer and concatenate layer. In the encoding layer, using BiLSTM [31] to process the input of the semantic vector, the work process is:

(3) $$\overset{↔}{x_{i}}=\overset{↔}{h}\left(T_{i},\;\overset{↔}{x_{i\pm 1}}\right),$$

(4) $$f\;\left(X\right)=\left[\overset{↔}{x_{1}},\overset{↔}{x_{i}},\ldots ,\overset{↔}{x_{i}}\right],$$

where $\overset{↔}{x_{i}}$ is the hidden state of $\vec{h}$ and $\overset{\leftarrow }{h}$, f (X) is the output of this encoding layer. The interaction layer mainly refers to the attention mechanism [32] and normalization. The output of the encoding layer is fed into the interaction layer to analyze the relationships between the words in the coded text and carry out the normalization of the text feature vectors through the attention mechanism:

(5) $$a\;\left(h_{i}\right)=\overset{k}{\underset{i=1}{\sum }}\frac{exp\left(w_{\alpha }^{T}\;f\left(X\right)\right)}{\sum_{b=1}^{k}A_{bj}}\;h_{j},$$

(6) $$a{\;}_{N_{i}}=\frac{a\;\left(h_{i}\right)}{\sqrt{\sum_{i=1}^{L}a\;{\left(h_{i}\right)}^{2}}}\;,$$

(7) $$I\left(T\right)=\left[a{\;}_{N_{1}},a{\;}_{N_{2}},\;\ldots ,\;a{\;}_{N_{i}}\right],$$

where $w_{\alpha }^{T}$ is the parameters that can be trained, $a\;\left(h_{i}\right)$ is the output of the attention. $a{\;}_{N_{i}}$ is the result from the normalization layer, then I(T) is the output of the single interaction layer, the final output of the concatenate layer is:

(8) $$\mathrm{Interaction}=[\mathrm{concatenate}\;(X,\;I\left(T\right)){]}^{\mathrm{OD}}$$

After conducting quantitative experiments, and taking various evaluation indexes of classification tasks as standards, the optimal depth (OD) inside the interaction layer model was set to six. After processing by the internal six-layer semantic extraction module, the output interaction is then be fed into the capsule module.

### 3.3. The Capsule Module (CM)

To date, text modeling methods are mainly of two types: one is conducting the shallow semantic modeling of text ignoring word order, and the other is conducting the deep semantic modeling of text considering the word order. For traditional deep neural networks, there is the problem of low model efficiency. The number of feature detectors to be copied or the number of labeled training data required by such methods increases exponentially with the data dimension. Spatial insensitive methods are inevitably limited by the effective encoding of rich text structures and lack of text expression ability as well. The capsule network uses the neuron vector to take the place of the single-neuron node of the traditional neural network and trains this new neural network in the way of dynamic routing, which effectively improves the above shortcomings [33].

A capsule network encodes two kinds of information, space data and presence probability, which are represented in the style of a capsule vector. It stands for the probability of the presence, and the attitude information is represented by the direction of the vector. The moving feature changes the capsule vector, but does not affect the feature presence probability. The capsule network mainly consists of the capsule internal operation and dynamic routing between the capsules. By inputting the vector matrix X, the capsule network encodes the space link from high-level features to low-level features. The weight update is implemented through dynamic routing to decide on the capsule that should be the next processing individual and weighted sum. The vector is compressed using squash, so that the length is between 0 and 1 and the direction remains the same. The Algorithm 1 shows our proposed model workflow with the capsule network for short text classification.
**Algorithm 1:** Short text classification algorithm based on the capsule and multi-level semantic extraction.**Input:** Short text data  **Output:** The probability distribution of the classification category.1: Data preprocessing.2: Embed each short text data then obtain M = [$m_{1}$, $m_{2}$, …, $m_{i}$].3: Input short text into the convolution layer for feature extraction, then obtain the feature mapping X = [$x_{1}$, $x_{2}$, …, $x_{i}$].4: Input the original short text and convolution features into the multi-layer semantic feature module and define the optimal depth parameter:    **for** r=optimal depth **do**       The input passes through the encoding layer, interaction layer, concatenate layer     **return** *Interaction*    **for**  i=length of Interaction **do**       Feed the input to the convolution layer and obtain the output $M=\left[m_{1},m_{2},\ldots ,m_{N}\right]$       Connect with a capsule layer by the dynamic routing algorithm            ${\hat{u}}_{j|i}=W_{ij}m_{i}$      **Algorithm** ROUTING (${\hat{u}}_{j|i},\;r,l$)       begin         for all capsule *i* in layer l and capsule j in layer (l+1): $b_{ij}\leftarrow 0$.          **for** r iterations **do**
         for all capsule i in layer l: $c_{i}\leftarrow softmax\left(b_{i}\right)$         for all capsule j in layer (l+1): $s_{j}\leftarrow \underset{i}{\sum }c_{ij}{\hat{u}}_{j|i}$         for all capsule j in layer (l+1): $v_{j}\leftarrow squash\left(s_{j}\right)$         for all capsule i in layer l and capsule j in layer (l+1): $b_{ij}\leftarrow b_{ij}+{\hat{u}}_{j|i}\cdot v_{j}$       **return** $v_{j}$       End       Calculate the probability distribution of the classification category.             **end for**

The convolution layer uses various convolution filters to extract semantic features from the different positions of sentences. The input of every short text is represented as x∈ℝ; *x_i_* is the *i*th word vector of the short text vector matrix. *W^a^* is the convolution operation filter. Each filter generates a column feature map $m_{i}^{a}$ at every location of the word window $x_{i:i+K_{l}-1}$. Each element $m_{i}^{a}$ in the feature set is given by the following formula:

(9) $$m_{i}^{a}=f\left(x_{i:i+K_{l}-1}\circ W^{a}+b_{0}\right),$$

where ∘ represents the cell multiplication, $b_{0}$ is the offset item, and f is a nonlinear activation function. For a=1,…,N, a total of N filters can produce N feature maps, and the final arrangement is:

(10) $$M=\left[m_{1},m_{2},\ldots ,m_{N}\right],$$

In the primary capsule layer, vector output capsules were used to replace CNN’s scalar output feature detector to preserve the local order and semantic representation of the instantiation parameters. There is a window to slide, each N-gram vector represents $M_{i}$ for each matrix multiplication, and produces the corresponding N-gram phrase in capsule form. Filter $W^{b}$ multiplies $M_{i}$ step by step to produce a set of capsules P; the capsule $p_{i}$ in P is calculated as:

(11) $$p_{i}=g\left(W^{b}M_{i}+b_{l}\right),$$

where g() is the compression function, $b_{l}$ is the capsule bias term. For all N filters, the produced capsule feature map is presented as:

(12) $$P=\left[p_{1},p_{2},\ldots ,p_{N}\right],$$

**Figure 4:**
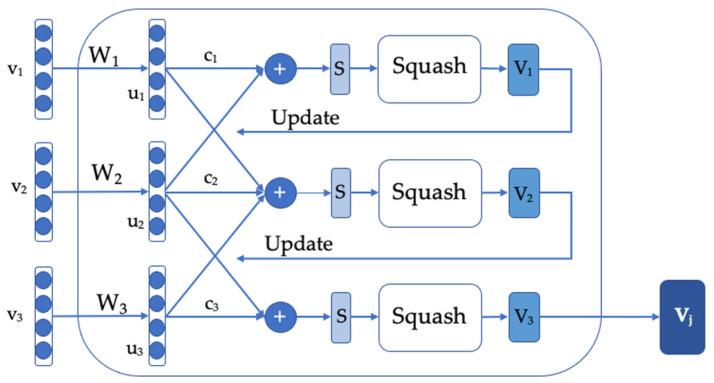
Dynamic routing structure in our model (n_routing_ = 3).

The parameters, such as $W^{b}$, in the capsule network are updated by the dynamic routing algorithm. The structure of it in our framework has been listed in Figure 4, which sets the number of routing as 3. First of all, obtain the prediction vector ${\hat{u}}_{j|i}$, define the number of iterations r and the current input capsule, which belongs to the lth layer of the network. For the lth layer of all the input *i* and output capsules *j*, define an initial weight parameter $b_{ij}$, which will be initialized as 0. Calculate the value of vector $c_{i}$, which is all the routing weights of capsule *i*. Note that the SoftMax function is used to ensure $\sum_{j}c_{ij}=1$:

(13) $$s_{j}=\underset{i}{\sum }c_{ij}{\hat{u}}_{j|i},$$

The weighted sum of the predicted vectors is performed using the above formula. The vector in the last step is guaranteed to remain unchanged by nonlinear Squash, but its length is forced not to exceed 1. After the final vector $v_{j}$ is output, the new weight value is updated by the following formula:

(14) $$b_{ij}=b_{ij}+{\hat{u}}_{j|i}\cdot v_{j},$$

The dot product operation at this point is to detect the similarity between the input and output of the capsule. Then, update the weight by carrying out the next iteration. After r iterations, the final output vector $v_{j}$ is returned.

## 4. Experimental Procedure and Results

### 4.1. Datasets

This mainly includes four public datasets, namely, SUBJ, TREC, MR and ProcCons.

**SUBJ:** The subjectivity dataset contains all the data used for text classifications, with a consideration of the subjective or objective of a sentence, which was first expressed in [34]. This was obtained from Rotten Tomatoes (http://www.rottentomatoes.com/ accessed on 29 September 2004) and presents snippets of movie reviews and plots summaries from movies from the IMDB database. 

**TREC:** The first time this was used was is in [35]. It mainly consists of questions and six question types. The information of these questions concerned the person, location and numeric information. This dataset contains all the data for the text classification task, which includes training and testing question data and the definition of question class. The main five classes include abbreviation (ABBR); entity, such as animal/body (ENTY); description, such as definition/manner/reason (DESC); human, such as group/individual(HUM); location, including city/country (LOC); and number, such as code/date/count (NUM).

**MR:** Movie Review is a dataset that summarizes each sentence in a review document, each sentence containing a subjective label for the overall opinion (positive or negative) in the review document. This dataset was introduced in the Proceeding of EMNLP 2002 [36] and is still used today in text classification work. MR contains a total of 10,662 sentences, including 5331 positive sentences and 5331 negative sentences. 

**ProcCons:** It is a dataset from the website (https://www.cs.uic.edu/liub/FBS/sentiment-analysis.html accessed on 15 April 2019) and it mainly contains short text, which is used for determining context-dependent sentiment words.

Table 1 represents the main information of four datasets, DS represents the dataset size, and ASL represents the average sentence length. For testing the performance of our capsule-based multi-level semantic framework, the dataset was divided into three types: 75% for training, 15%, and 15% for validation testing, respectively.

### 4.2. Model Configurations

The system used in this work is Ubuntu 20.04.2 LTS, the graphics card is Nvidia GeForce RTX 3070 with 8G memory, the CUDA version is 11.0, the programing language is python 3.7, and the deep learning framework is TensorFlow. The selected optimizer was Adam. To perform testing with the model, the parameter number of routing for the capsule was 3.

### 4.3. Baseline Methods

In order to objectively evaluate our model, we compared it to some other state-of-the-art models. The baseline networks are introduced as follows:

**CNN for SC** [37]: a classifier based on CNN by applying the convolution operation.

**LR-Bi-LSTM** [38]: a model applies Bi-LSTM based on a linguistic regularizer.

**VA LSTM** [39]: a framework adds perturbations to the word embedding to strengthen the model robustness and improve the accuracy of the classifier.

Bi-BloSAN* [40]: uses attention to compress the output of bi-directional block self-attention into a vector representation. The model has high parallelism and good modeling of local and remote correlations.

**TE-LSTM+c,p** [41]: a model combined with the semantic information of phrases, with a consideration of POS tags to apply to the gates of the tree-structured LSTM.

**Transformer** [42]: uses stacked self-attention blocks to learn semantic dependency.

**AGN** [43]: a model that merges statistical features and uses a valve mechanism to train a robust classifier to improve the performance of text classification.

**TextING** [44]: a model that improves the representation of the contextual word relationship within a document by GNN.

**VGCN-Bert** [45]: combines Bert with vocabulary GCN to improve the representation of local and global information.

**HGAT** [46]: models heterogeneous information for short text and combines node-level and type-level attention to improve the performance of short text classification.

**MP-GCN** [47]: focuses on node representation learning by multi-head pooling GCN without the help of pre-training word embedding.

**CapsNet (DR)/CapsNet (EMR)**: Dynamic Routing (DR), Expectation and Maximization Routing (EMR) are two methods to group the capsules and produce the parent capsules, then calculate the output of the capsules.

### 4.4. Exerimental Results

Our experiment result shows that the accuracy of the capsule-based multi-level semantic extraction model is higher than the capsule network with DR and EMR, which is displayed in Table 2. The main reason for this can be concluded as the added convolution module and our multi-level semantic extraction module, which improves the utilization of the eigenmatrix of the model to the greatest extent. From the quantitative experiment results for the comparison models (i.e., LSTMs and Transformer) in Table 2, our model outperforms other models in terms of accuracy, for SUBJ, TREC and ProcCons. For MR, except for VA LSTM [32], our models attain the best results, compared to the rest of the models. However, the results of the other three datasets are all greater than for VA LSTM [32].

Table 3 indicates the results of the four datasets. Three super parameters were explored for our model, which are the depth of the multi-level semantic extraction, embedding size and the epoch. The other relevant parameters are illustrated in Table 4. The description is as follows: DME means depth of multi-level semantic extraction, ES represents embedding size, E is epoch, and BS demonstrates batch size. The selection of a suitable depth is important because the sparsity of short text and the running speed of the model must be considered. From Table 3, we can conclude that the best results are attained for four datasets in the depth of six, and this can be regarded as the most suitable number for our model. The best result comes from the embedding sizes 20, 20, 200 and 200 for SUBJ, TREC, MR and ProcCons, respectively.

### 4.5. Discussion

#### 4.5.1. Ablation Study

Table 5 mainly focuses on emphasizing the effectiveness of our framework. In Table 5, CSMM represents the convolution semantic matrix module, and MlSEM means the multi-level semantic extraction module. As mentioned in the paper, after a lot of experiments were performed using this model, it was determined that the most suitable depth of the multi-layer feature extraction for this model was six. Additionally, the results of the corresponding experiments are the direct source of evidence to verify the validity of the model proposed in this paper. In addition, “-” represents the deletion of the relevant modules in this model, and “+” represents the addition of some modules on the basis of this model, which is also the main method used to prove the stability of the model. 

From the following ablation experiments, we found that each module of our proposed model contributes to the performance. From the results of the structure of -CSMM, -MlSEM, and -CM, it can be observed that the influence of the MlSEM is higher than CSMM and CM, since the results decrease more than the results of the other two structures, especially on the datasets of SUBJ and TREC. The single module of CM has the worst results on the datasets of SUBJ and TREC, while the single module MlSEM has the worst results on datasets MR and ProcCons. The last two structures, which are -CSMM+(D-MlSEM) and -CSMM-MlSEM+BiGRU, are aims to evaluate the part of the proposed model that presents the best result by combining the CM module. Therefore, D-MllSEM and BiGRU were applied as the compared structure. From the results of -CSMM+(D-MlSEM) and -CSMM, we can conclude that our proposed model presents a better result for SUBJ, MR and ProcCons, while presenting a small gap on TREC. The results of -CSMM-MlSEM+BiGRU and -CSMM attained a similar conclusion for MR and ProcCons, while MlSEM+CM also had a better performance than BiGRU+CM on TREC.

#### 4.5.2. The Depth of the MlSEM Study

**Figure 5:**
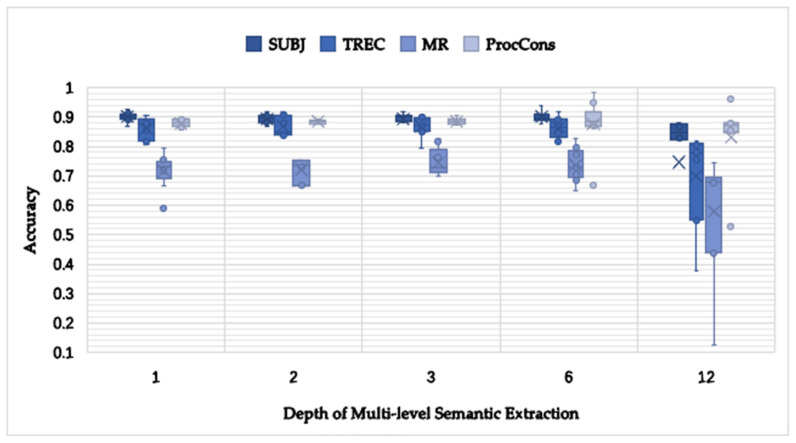
Accuracy results with the depth change of multi-level semantic extraction using the four datasets.

In order to find the most suitable depth of MlSEM, Figure 5 shows the accuracy results with the depths of 1, 2, 3, 6 and 12. It illustrates that the accuracy of the change range in depths of 1, 2, 3 and 6 is smaller than that in the depth of 12. The trend of classification accuracy is up when the depth increases. However, it lowers when the depth is 12. As a result, the capacity to show the knowledge of the semantic becomes greater when training a vector, and it changes to represent a various feature of the semantic, whereas a moderate depth exists. 

At the same time, the model produced the best results in the depth of six and it had better accuracy in all four datasets, compared to that in the depth of two. As there is often a turning point, transition and other statements in the short text, it is necessary to dig into the content of the text in-depth and elaborate on this. MlSEM processes both forward and backward text semantics, making it possible for the model to learn more hidden information, distinguish important information and enhance the semantic expression ability. The in-depth exploration of MlSEM can improve the semantic extraction capacity of the existing shallow short text classification model to a certain extent, and extract semantic information from multiple levels and aspects, thus improving the short text classification result.

#### 4.5.3. The Effect of Routing in CM

**Figure 6:**
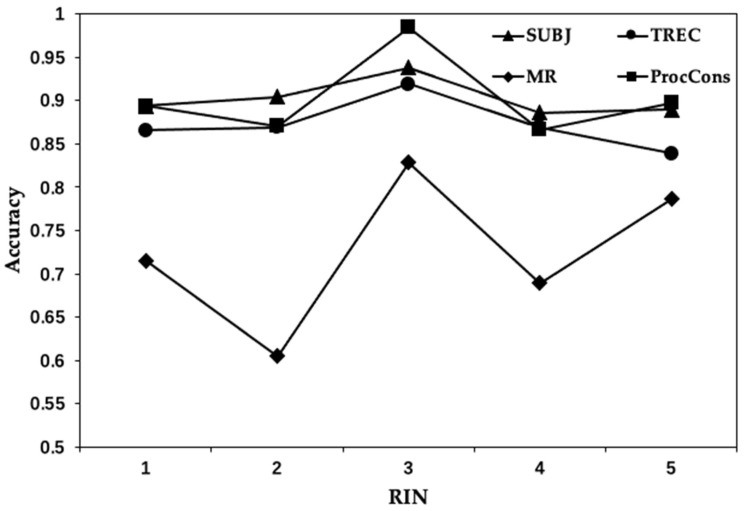
Accuracy results with the variations of the routing iteration number (RIN).

As our proposed model includes an iterative process during routing, the parameter setting of it is also a significant part. Therefore, we explore the performance of the model in Figure 6, according to the variation of routing iteration number (RIN), but keeping the number of trainable parameters. We conducted quantitative experiments using datasets SUBJ, TREC and MR, and ProcCons with varying RIN from 1 to 5. From Figure 6, we can conclude that our model achieves the best result when RIN is set to three on the dataset SUBJ, TREC, MR and ProcCons. When RIN is 1, our capsule network acts as a standard network structure. While increasing RIN, the performance becomes dramatically worse, especially on the dataset of TREC. Moreover, as the RIN increases to five, the training process of the model becomes more difficult. The change range of the results becomes greater, which means that the model becomes unstable under this condition. As a result, it is necessary to restrict the RIN according to the performance.

## 5. Conclusions

In this paper, we proposed a novel capsule-based multi-level semantic extraction model for short text classification. It parses short text semantics from multiple channels and effectively improves the performance of short text classification. Feature multi-level extraction for short text can not only help in short text classification, but also other tasks, such as short text similarity and short text paraphrase identification [48]. In this model, the convolutional encoded text vector, BiLSTM, normalization and attention were used to obtain the remote dependencies of text information captured in the pre-hidden and post-hidden layers of short texts to form new features, and the high-level local features of text semantics were obtained through capsule networks and the dynamic routing mechanism. The comparative experiments of four common datasets, including text sentiment analysis and the multi-classification task, prove that the model has a certain robustness and generalization ability. In addition, this paper also studied the influence of the multi-level short text semantic extraction depth on classification results and obtained the optimal depth for short text semantic extraction through experimental exploration, which improved the results for the four datasets. In the future, we will not only study the capsule-based multi-level semantic extraction model for short text classification, but also explore the unified language representation of short text from the form of semantic representation of short text and apply it to various tasks to study its impact.

## Figures and Tables

**Table 1:** Information about the four datasets.

Dataset	Type	Classes	DS	ASL
SUBJ	Snippets of movie reviews	2	10,000	13
TREC	Question	6	5952	5
MR	Review sentences	2	10,662	10
ProcCons	Short text	2	45,875	6

**Table 2:** The accuracy for the four datasets.

Model	SUBJ	TREC	MR	ProcCons
**CNN for SC** [37]	0.9000	0.9120	0.8110	-
**LR-Bi-LSTM** [38]	0.9022	0.9134	0.8222	0.9694
**VA LSTM** [39]	0.9110	-	0.8340	0.9765
Bi-BloSAN * [40]	-	0.9100	0.7966	-
**TE-LSTM_+c,p_** [41]	0.8878	0.9024	0.8220	0.8989
**Transformer** [42]	0.8803	0.8738	0.8190	0.9683
**Transformer +AGN** [43]	0.8897	0.8885	0.8222	0.9667
**TextING** [44]	0.9029	0.7832	0.7790	0.9465
**VGCN-BERT** [45]	0.9013	0.8982	**0.8666**	0.9667
**HGAT** [46]	0.8347	0.7072	0.6273	-
**MP-GCN** [47]	0.9117	0.7980	0.7802	0.9479
**CapsNet (EMR)**	0.8070	0.6658	0.5787	-
**CapsNet (DR)**	0.8900	0.7650	0.7300	0.9163
**Ours**	**0.9380**	**0.9194**	**0.8281**	**0.9843**

**Table 3:** The results of the four datasets with three parameters of the variable by our approach.

Depth	Embed	Epoch	SUBJ	TREC	MR	ProcCons
6	20	5	0.9020	0.8187	0.6484	0.8818
		10	0.8980	0.8187	0.7958	0.8588
		15	0.8940	0.8691	**0.8281**	0.6673
		20	0.8920	0.8456	0.7059	0.9475
		25	0.9100	0.8657	0.7453	**0.9843**
	200	5	**0.9380**	0.8557	0.7734	0.8918
		10	0.8840	0.8926	0.6835	0.8884
		15	0.9100	**0.9194**	0.7228	0.8862
		20	0.8759	0.8926	0.7284	0.8748
		25	0.8980	0.9194	0.7340	0.8801
12	20	5	0.1039	0.3791	0.1235	0.8827
		10	0.8760	0.7550	0.4382	0.8718
		15	0.8799	0.8120	0.6966	0.9607
		20	0.8299	0.8053	0.6797	0.8801
		25	0.8459	0.7785	0.6966	0.5274
	200	5	0.1260	0.5503	0.7434	0.8500
		10	0.8240	0.8187	0.6760	0.8526
		15	0.8300	0.8624	0.6610	0.8823
		20	0.8359	0.8859	0.7097	0.8731
		25	0.8680	0.8187	0.6985	0.8700

**Table 4:** Hyperparameter settings.

Dataset	DME	ES	E	BS
SUBJ	6	200	5	100
TREC	6	200	15	100
MR	6	20	15	100
ProcCons	6	20	25	100

**Table 5:** Ablation experiment of our proposed model on the four datasets. The experiment analyzes the performance comparison when using different combinations of the three modules (CSMM, MlSEM and CM).

Structure	SUBJ	TREC	MR	ProcCons
**Ours**	**0.9380**	**0.9194**	**0.8281**	**0.9843**
-CSMM	0.9019	0.8288	0.7930	0.8905
-MlSEM	0.8699	0.7214	0.7303	0.8805
-CM	0.9019	0.9026	0.7340	0.8809
-CSMM-MlSEM	0.8559	0.7281	0.7322	0.8757
-CSMM-CM	0.8679	0.8590	0.7059	0.8630
-MlSEM-CM	0.8620	0.8523	0.7303	0.8740
-CSMM+ (D-MlSEM)	0.8760	0.8691	0.6947	0.8731
-CSMM-MlSEM+BiGRU	0.9120	0.6174	0.6516	0.8857

## Data Availability

Not applicable.
